# The spectrum of rare central nervous system (CNS) tumors with *EWSR1*‐non‐ETS fusions: experience from three pediatric institutions with review of the literature

**DOI:** 10.1111/bpa.12900

**Published:** 2020-11-06

**Authors:** Oscar Lopez‐Nunez, Barbara Cafferata, Mariarita Santi, Sarangarajan Ranganathan, Thomas M. Pearce, Scott M. Kulich, Kelly M. Bailey, Alberto Broniscer, Sabrina Rossi, Angelica Zin, MacLean P. Nasrallah, Marilyn M. Li, Yiming Zhong, Evelina Miele, Rita Alaggio, Lea F. Surrey

**Affiliations:** ^1^ Department of Pathology and Laboratory Medicine University of Pittsburgh Medical Center Pittsburgh PA; ^2^ Department of Pathology and Laboratory Medicine Cincinnati Children’s Hospital Medical Center Cincinnati OH; ^3^ General Pathology and Cytopathology Unit Department of Medicine‐DIMED University of Padova Padova Italy; ^4^ Department of Pathology and Laboratory Medicine Perelman School of Medicine at the University of Pennsylvania Philadelphia PA; ^5^ University of Pittsburgh School of Medicine Pittsburgh PA; ^6^ Division of Neuropathology Department of Pathology University of Pittsburgh Medical Center Pittsburgh PA; ^7^ Division of Pediatric Hematology/Oncology University of Pittsburgh School of Medicine Pittsburgh PA; ^8^ Department of Pathology Bambino Gesù Children’s Hospital IRCCS Rome Italy; ^9^ Institute of Pediatric Research (IRP) Fondazione Città della Speranza Padova Italy; ^10^ Department of Pediatric Onco‐Hematology and Cell and Gene Therapy Bambino Gesù Children’s Hospital IRCCS Rome Italy

**Keywords:** CNS, *CREB1*, *CREM*, *EWSR1*‐non‐ETS fusions, *PATZ1*, pediatric, *PLAGL1*, *WT1*

## Abstract

The group of CNS mesenchymal (non‐meningothelial) and primary glial/neuronal tumors in association with *EWSR1‐non‐*ETS rearrangements comprises a growing spectrum of entities, mostly reported in isolation with incomplete molecular profiling. Archival files from three pediatric institutions were queried for unusual cases of pediatric (≤21 years) CNS *EWSR1*‐rearranged tumors confirmed by at least one molecular technique. Extra‐axial tumors and cases with a diagnosis of Ewing sarcoma (*EWSR1*‐ETS family fusions) were excluded. Additional studies, including anchored multiplex‐PCR with next‐generation sequencing and DNA methylation profiling, were performed as needed to determine fusion partner status and brain tumor methylation class, respectively. Five cases (median 17 years) were identified (M:F of 3:2). Location was parenchymal (n = 3) and undetermined (n = 2) with topographic distributions including posterior fossa (n = 1), frontal (n = 1), temporal (n = 1), parietal (n = 1) and occipital (n = 1) lobes. Final designation with fusion findings included desmoplastic small round cell tumor (*EWSR1‐WT1*; n = 1) and tumors of uncertain histogenesis (*EWSR1‐CREM*, n = 1; *EWSR1‐CREB1*, n = 1; *EWSR1‐PLAGL1*, n = 1; and *EWSR1‐PATZ1*, n = 1). Tumors showed a wide spectrum of morphology and biologic behavior. For *EWSR1‐CREM*, *EWSR1‐PLAGL1* and *EWSR1‐PATZ1* tumors, no significant methylation scores were reached in the known brain tumor classes. Available outcome (4/5) was reported as favorable (n = 2) and unfavorable (n = 2) with a median follow‐up of 30 months. In conclusion, we describe five primary *EWSR1*‐non*‐*ETS fused CNS tumors exhibiting morphologic and biologic heterogeneity and we highlight the clinical importance of determining specific fusion partners to improve diagnostic accuracy, treatment and monitoring. Larger prospective clinicopathological and molecular studies are needed to determine the prognostic implications of histotypes, anatomical location, fusion partners, breakpoints and methylation profiles in patients with these rare tumors.

## Graphical abstract

Pediatric EWSR1‐non‐ETS tumors of the CNS are rare and have not been addressed comprehensively, apart from conventional Ewing sarcoma. Here, we characterize the pathological and molecular features of five such tumors.

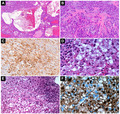

## Introduction

The group of mesenchymal (non‐meningothelial) tumors of the central nervous system (CNS) comprises various entities thought to arise predominantly from meninges and surrounding bone structures. However, and less frequently, primary intraparenchymal and choroid plexus tumors can be seen. Overall, these neoplasms are histologically similar to their extracranial bone and soft tissue counterparts (40).

In pediatric patients, the *EWSR1*‐rearranged mesenchymal tumors of the CNS encompass a rare and heterogeneous group of entities mainly represented by Ewing sarcoma but also isolated myoepithelial neoplasms, unusual desmoplastic small round cell tumors (39) and more recently, by the poorly defined group of intracranial lesions involving members of the cAMP response element‐binding protein (CREB) family (10, 25, 33, 38, 86). The *EWSR1* gene (22q12.2) is well‐known as a “promiscuous” transcript, and not infrequently, identical gene fusions can be shared by multiple tumor entities (5, 86). As the growing gamut of these lesions unveils, the sole identification of an *EWSR1* rearrangement by FISH is no longer sufficient, requiring further identification of fusion partner genes and morphologic correlation for correct diagnosis (29).

Most primary *EWSR1*‐rearranged tumors of the CNS have been reported either in isolation or in more extensive series including a broad range of age groups, often with incomplete molecular profiling and unknown fusion partners. Our goal is to characterize further the morphologic spectrum and fusion partners of rare *EWSR1*‐non‐ETS‐rearranged tumors of the CNS in a cohort of cases collected from three large pediatric institutions.

## Materials and Methods

Institutional and consultation records from three large pediatric institutions (Children's Hospital of Philadelphia, Pennsylvania, USA; UPMC Children's Hospital of Pittsburgh, Pennsylvania, USA, and Bambino Gesù Children's Hospital, Rome, Italy) were searched for brain or spinal cord *EWSR1*‐rearranged neoplasms diagnosed over 20 years; following institutional review board approval. Only patients (≤21 years of age) with intracranial CNS tumors harboring an *EWSR1* rearrangement confirmed by at least one molecular technique were selected, regardless of the original diagnosis. The pertinent clinical information was retrieved from electronic medical records and available consultation material with emphasis on clinical features at presentation as well as presurgical and postsurgical imaging studies. Cases where tumors arose primarily from cranial bones were excluded from further analysis. In addition, parenchymal (intra‐axial) tumors with classic features of Ewing sarcoma including morphology, immunohistochemistry and ETS‐family fusion genes (eg, *EWSR1‐FLI1*) were also excluded given their extensive description in the literature.

Hematoxylin and eosin‐stained sections and available immunohistochemical and molecular material were retrieved and reviewed by three pediatric pathologists and four neuropathologists. The histologic pattern, cytologic features, presence or absence of atypia and stromal characteristics were documented for each case. Additional immunohistochemical, molecularand/or FISH studies for *EWSR1* rearrangements were conducted as needed. Immunohistochemistry was performed per standard protocols at each institution. A list of antibodies with corresponding technical information is summarized in Table S1.

FISH for *EWSR1* rearrangements was performed using a break‐apart probe according to standard protocols (85). Custom targeted anchored multiplex PCR was performed, followed by next‐generation sequencing (NGS) (Archer DX, Boulder, CO), as previously described (15). Briefly, RNA or total nucleic acid was extracted from fresh frozen or formalin‐fixed, paraffin‐embedded tissue, respectively. RNA was reverse‐transcribed to generate cDNA and molecular barcode adapters were ligated to cDNA followed by two rounds of target‐specific PCR. All libraries were sequenced on Illumina HiSeq (Illumina, San Diego, CA). Data analysis was performed using Archer analysis software. In some instances, identified fusions were then confirmed with Sanger sequencing. DNA methylation profiling was performed on three of the tumors (cases 2, 3 and 4) as previously described (44, 51). Tumor areas with highest tumor cell content were selected for analysis. DNA was extracted according to MagPurix FFPE DNA Extraction Kit (Resnova, Rome, Italy) for automatic extraction of genomic DNA. Samples were analyzed using Illumina Infinium Human Methylation EPIC BeadChip (EPIC) arrays (Illumina, San Diego, USA) according to the manufacturer’s instructions, on Illumina iScan Platform (Illumina, San Diego, USA). Generated methylation data were compared to brain tumor classifier v11b4 (13) developed by Heidelberg University and DKFZ (https://www.molecularneuropatholog.org/mnp/classifier/all) to assign a subgroup score for the tumors in the known methylation classes.

## Results

### Clinical findings

Five primary CNS neoplasms harboring an *EWSR1*‐non‐ETS gene fusion were identified (Table 1). The median age at diagnosis was 17 years (range: 4–20 years) with a 3:2 male to female ratio. The specific location of tumors was documented as follows: frontal lobe (n = 1), temporal lobe (n = 1), parietal lobe (n = 1), occipital lobe (n = 1) and posterior fossa (n = 1). By imaging, tumors were parenchymal (n = 3) and undetermined (n = 2). All patients underwent whole‐body imaging at the time of diagnosis without any extracranial lesions and no evidence of skull involvement with subsequent intraparenchymal extension was identified, supporting their primary intracranial origin. On follow‐up (median: 30 months), no evidence of disease was documented in two cases and two patients are alive with recurrent disease (Cases 2 and 4). Follow‐up was lost in the remaining patient. Additional clinical findings are summarized in Table 1.

**Table 1:** Clinical, morphological and molecular features.

Case	Sex	Age (years)	Location (parenchymal vs. undetermined*)	Morphologic diagnosis	Fusion gene	Extent of surgical resection	Follow‐up (mo)	Outcome
1	M	17	Parietal lobe (undetermined)	Intracranial myxoid mesenchymal tumor	*EWSR1‐CREB1*	GTR	2	NED
2	F	20	Posterior fossa (parenchymal)	Intracranial tumor with AFH‐like features	*EWSR1‐CREM*	GTR	54	AWD
3	M	4	Frontal lobe (parenchymal)	Glioneuronal tumor, NEC	*EWSR1‐PLAGL1*	NTR	84	NED
4	F	19	Temporal lobe (undetermined)	Malignant, poorly differentiated neoplasm of the CNS	*EWSR1‐PATZ1*	NTR	5	AWD
5	M	6	Occipital lobe (parenchymal)	DSRCT	*EWSR1‐WT1*	STR	N/A	Unknown

Abbreviations: AFH = angiomatoid fibrous histiocytoma; AWD = alive with disease; CNS = central nervous system; DSRCT = desmoplastic small round cell tumor; GTR = gross total resection; N/A = not available; NEC = not elsewhere classified; NED = no evidence of disease; NTR = near‐total resection; STR = subtotal resection.

* For undetermined cases, intraparenchymal vs. extraparenchymal (meningeal) could not be determined by imaging.

### Pathologic, immunophenotypic and molecular findings

The average tumor size was 6.6 cm (range: 4.9 to 10 cm). Only one case demonstrated conventional small round cell morphology (Case 5). Two cases demonstrated features of mesenchymal neoplasms and were found to have CREB family fusions (Cases 1 and 2). One case showed glioneuronal differentiation (Case 3) while the final case was a poorly differentiation neoplasm of uncertain histogenesis (Case 4). Additional immunophenotypic features not described below are reported in Table S2. Detailed molecular findings for each case are summarized in Table S3 and Figures [Link], [Link], [Link].

#### EWSR1‐CREB family fused tumors

**Figure 1:**
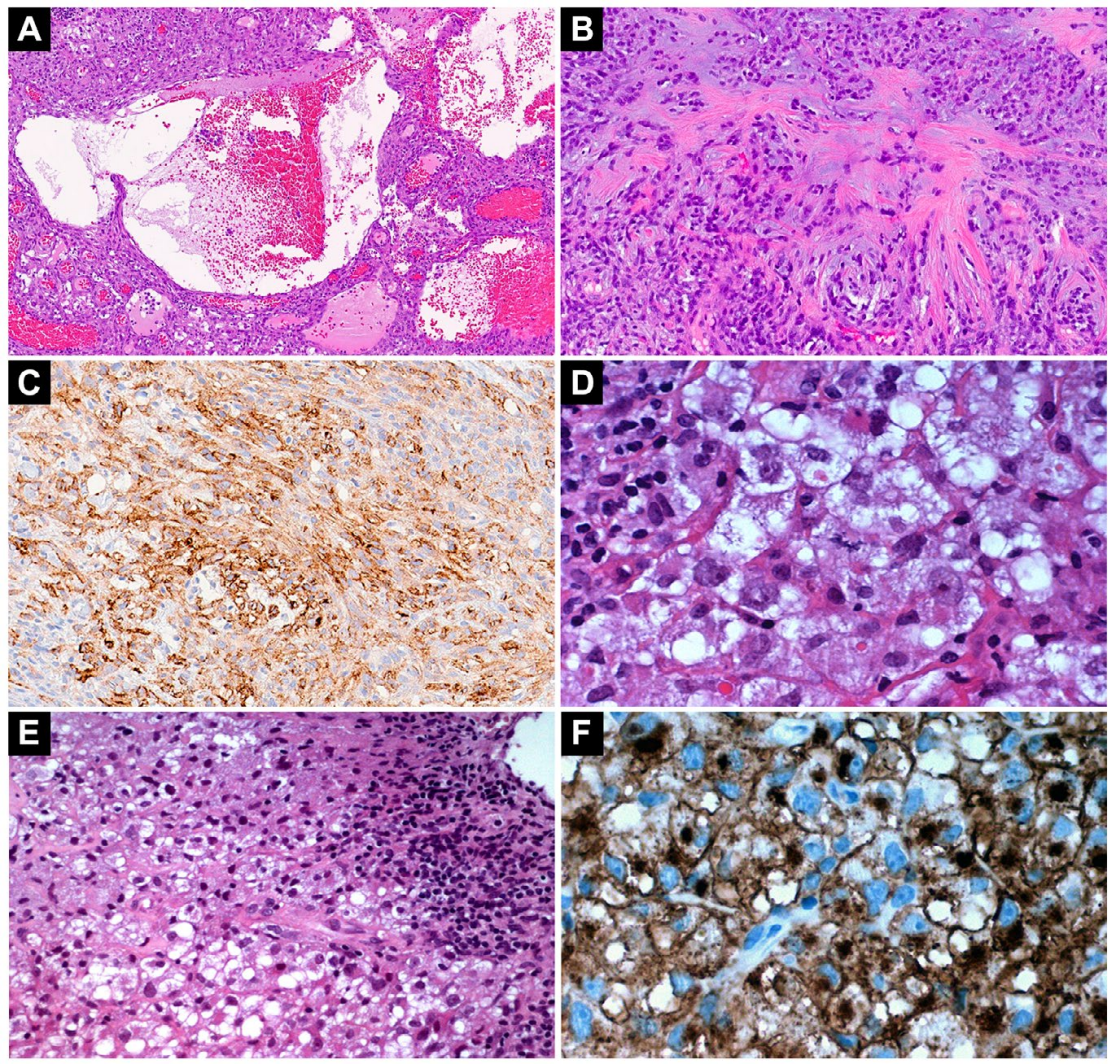
Morphology and immunophenotype of *EWSR1‐*rearranged tumors with CREB family fusion partners. Case 1, a myxoid tumor harboring an *EWSR1‐CREB1* fusion shows scattered blood‐filled pseudovascular spaces (**A**) and myxoid islands with amianthoid collagen deposition (**B**) showing immunoreactivity for EMA (**C**). Case 2, a tumor harboring an *EWSR1‐CREM* fusion shows diffuse sheets of epithelioid cells featuring large nuclei with prominent nucleoli (**D**) and a prominent peripheral lymphoplasmacytic infiltrate (**E**). The tumor cells are immunoreactive for GLUT‐1 with a cytoplasmic and prominent paranuclear/Golgi‐like (**F**) staining pattern.

Cases 1 and 2 were similar in that both harbored *EWSR1* gene fusions involving members of the CREB family (ie, *CREB1* and *CREM*) and showed an ambiguous immunophenotype (Figure 1). Case 1 was a myxoid mesenchymal tumor involving the parietal lobe. Microscopically, it was composed of sheets of cells with eosinophilic cytoplasm, ovoid to spindled nuclei and fine chromatin admixed with a prominent vascular component and scattered blood‐filled cystic spaces (Figure 1A). Several intervening areas with nodules containing cords of tumor cells within a variably myxoid stroma, amianthoid collagen fiber deposition (Figure 1B) and readily identifiable mitotic figures (up to 5 mitoses per high power field) were noted. Prominent perivascular lymphocytic infiltration was present at the periphery and the lesion was sharply demarcated from surrounding brain parenchyma featuring prominent reactive astrocytosis. The neoplastic cells showed patchy immunoreactivity for CD99 (membranous), GLUT‐1 (weak, cytoplasmic), S100 and epithelial membrane antigen (Figure 1C), while negative for smooth muscle actin, desmin, glial fibrillary acid protein and synaptophysin, among others. The Ki67 proliferation index was low (approximately 3%). SOX‐9 was negative. FISH studies were positive for *EWSR1* translocation with targeted RNA‐sequencing studies confirming an *EWSR1‐CREB1* rearrangement (Table 1).

Case 2 was an intra‐axial tumor arising from the posterior fossa composed of sheets of epithelioid cells with abundant eosinophilic to clear cytoplasm, large nuclei and prominent nucleoli (Figure 1D). There was brisk mitotic activity (up to 5 mitoses per 10 high power fields) without necrosis. At the periphery, a focal but prominent lymphoplasmacytic infiltrate was present (Figure 1E). No myxoid stroma or blood‐filled cystic spaces were evident in this lesion. The tumor cells were diffusely positive for CD99 (membranous), GLUT‐1 with a prominent paranuclear/Golgi pattern (Figure 1F) and focally positive for epithelial membrane antigen, glial fibrillary acid protein and synaptophysin while negative for desmin, OLIG2 and melanocytic markers (see also Table S2). The Ki67 proliferation index was high (approximately 50%). FISH studies were positive for *EWSR1* rearrangement with targeted RNA‐sequencing studies confirming *EWSR1‐CREM* rearrangement (Table 1 and Figure 3). Recently, DNA methylation profiling has proven to be a highly robust and reproducible approach for the classification of brain tumors and sarcomas into different diagnostic groups (13, 36). Global DNA methylation profiling did not reach significant scores for Case 2 in the currently identified CNS tumor classes. Copy number variation analysis showed loss of chromosomes 9 (including *CDKN2A* and *CDKN2B*), 13 and 14, as well as chromosome 10p deletion, possibly the result of the *EWSR1‐CREM* rearrangement (Figure S1A). Chromosome 22q losses were present but did not involve *EWSR1* (Figure S1B). The patient relapsed 2 years after the original diagnosis with local involvement of the transverse sinus and a histologically confirmed pelvic bone metastasis.

#### EWSR1‐PLAGL1 fused tumor

**Figure 2:**
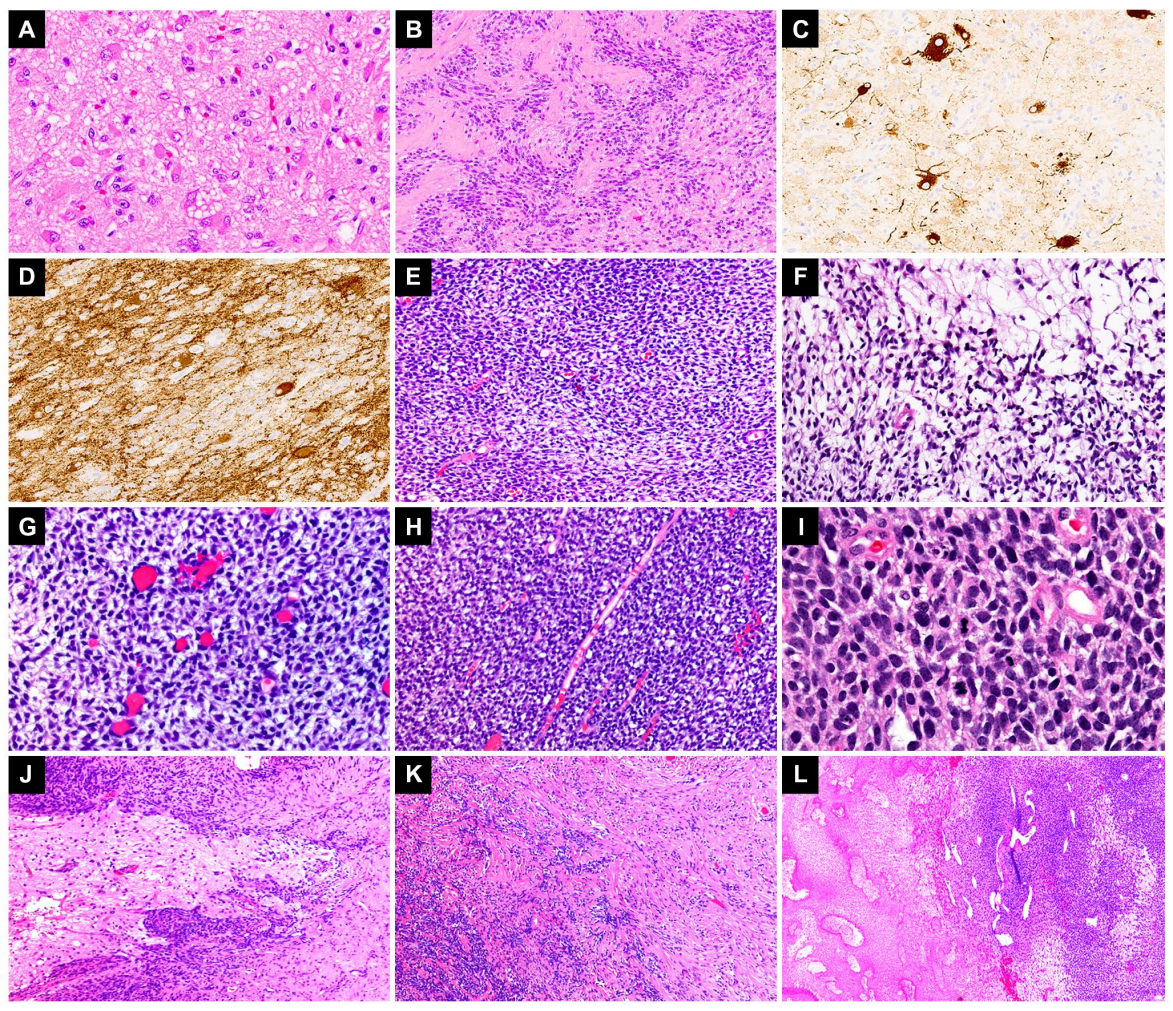
*EWSR1*‐rearranged tumors with biphasic morphology. Case 3, a glioneuronal tumor harboring an *EWSR1‐PLAGL1* fusion shows areas containing large vacuolated and multinucleated ganglion cells (**A**) admixed with round‐to‐spindled cell areas with palisading (**B**). The ganglion cells show diffuse immunoreactivity for Neurofilament protein **(C)** and Synaptophysin (**D**). Case 4, a poorly differentiated malignant neoplasm harboring *EWSR1‐PATZ1* fusion shows cells with clear cytoplasm (**E**,**F**) and interspersed vessels (**G**) admixed with sheets of monotonous, round‐to‐spindled cells with high nuclear‐to‐cytoplasmic ratio (**H, I**). Other areas showing interface of tumor cells with surrounding brain parenchyma **(J)**, dense collagenous stroma (**K**) and infarct‐type necrosis with staghorn gaping vessels (**L**) are shown.

**Figure 3:**
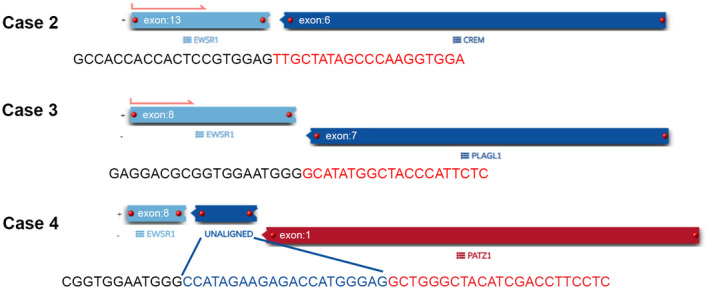
Examples of *EWSR1* breakpoint sequences obtained from anchored multiplex PCR from Cases 2, 3 and 4. Black sequence = *EWSR1*, Red sequence = fusion partner and Blue sequence (Case 4) = linker sequence (21 base pairs) which maintains reading frame.

Case 3 exhibited the features of a glioneuronal tumor with a variable biphasic pattern. The first pattern showed collections of large cells with copious and slightly basophilic cytoplasm, some clearly ganglionic in appearance with open vesicular nuclei and nucleoli. Some of these cells showed multinucleation or cytoplasmic vacuolization (Figure 2A). The second histologic component consisted of small groups and larger sheets of rather uniform cells with round nuclei, speckled chromatin and open cytoplasm forming in some areas striking palisaded arrays separated by thin‐walled capillaries (Figure 2B). Scattered mitoses are present in these areas, but no vascular proliferation or necrosis. In some areas, these two histologic components appeared more intermixed, whereas in other regions, they were more distinctly separate. Rosenthal fibers and eosinophilic granular bodies were not present. Occasional thin perivascular lymphocytic cuffs and numerous calcospherites were also identified. A panel of immunostains revealed strong immunoreactivity for neurofilament protein and synaptophysin in the ganglion cell‐like areas (Figure 2C,D). The small cell components demonstrated strong expression of GFAP; however, neurofilament protein was negative. Additionally, this component expressed a nonspecific immunophenotype (patchy positivity for S100 and epithelial membrane antigen with negative CD99). The proliferation index in small‐cell areas was approximately 5% to 10%. Both INI‐1 and BRG‐1 immunostains were preserved. FISH studies were positive for *EWSR1* translocation and targeted RNA‐sequencing studies demonstrated *EWSR1‐PLAGL1* rearrangement (Table 1 and Figure 3). This tumor did not classify into any of the known brain tumor methylation classes. The copy number plot identified a loss of chromosomal material corresponding to the breakpoints of *PLAGL1* on 6q24 (Figure S2). The patient was treated with surgical resection without further intervention and is currently alive without evidence of disease after 84 months of follow‐up.

#### EWSR1‐PATZ1 fused tumor

An additional temporal lobe tumor, Case 4, showed a biphasic malignant lesion composed of areas of cells with clear cytoplasm admixed with sheets of monotonous, round to spindled cells (Figure 2E,F). Some areas showed tumor cells embedded in a dense collagenous stroma. Brisk mitotic activity, geographic necrosis and numerous apoptotic bodies were also noted. The tumor appeared well‐circumscribed with only focal infiltration into surrounding brain parenchyma and meningeal involvement; however, an intra vs. extra‐axial origin was difficult to determine. A comprehensive panel of immunohistochemical stains was conducted, revealing a widely negative immunophenotype (Table S2). The Ki67 proliferation index was approximately 70% in the most active foci. FISH studies were negative for rearrangements in *EWSR1*, *SYT*, *FUS*, *NUT*, *CIC* and *BCOR*; however, an *EWSR1‐PATZ1* fusion gene was ultimately confirmed by NGS studies (Table 1 and Figure 3). Similarly to the previous cases, the tumor did not match in any methylation classes of the brain tumor classifier. Copy number variation plot revealed the loss of partial chromosome 15q, 21q and 22q, as well as partial 1q gain and a complex rearrangement of chromosome 19 with C19MC loss. Notably neither *EWSR1* nor *PAZT1* were included in the deleted chromosomal 22q region (Figure S3). Following near‐total resection, the patient underwent a course of proton beam irradiation (total dose 5940 cGy in 180 cGy fractions) followed by radiosurgery (total dose 1200 cGy in 400 cGy fractions) because of recurrence. The tumor has been treated as a high‐grade sarcoma despite its uncertain histogenesis and the patient is alive with disease at 5 months following surgical resection.

#### EWSR1‐WT1 fused tumor

This tumor was located in the left occipital lobe, exhibiting sheets of small round cells with scant cytoplasm and vesicular chromatin alternating with large areas of clear cells and minimal stromal desmoplasia (Case 5). The specific site of origin was deemed to be parenchymal by imaging studies. Both INI‐1 and BRG‐1 immunostains were preserved. The *EWSR1* rearrangement was determined by FISH, which also showed negative rearrangements involving C11 or f95 and *RELA*. Subsequent reverse transcription‐polymerase chain reaction studies were positive for *EWSR1‐WT1* fusion and negative for *EWSR1‐FLI1* and *EWSR1‐ERG* transcripts. No follow‐up information was available for this patient.

## Discussion

*EWSR1* is known for its propensity to translocate to a variety of fusion partners characteristically present in a gamut of mesenchymal tumors, sometimes featuring disparate morphologies and clinical behavior (71). As this study and others demonstrate, there is growing evidence indicating *EWSR1* involvement in primary, non‐mesenchymal CNS neoplasms (65). There has been an increasing shift in pathology practice such that the sole identification of *EWSR1* rearrangement by FISH is no longer considered sufficient but oftentimes requires further ancillary studies to determine fusion partner genes along with morphologic correlation for correct diagnosis (5, 58). Thus, the presence of an *EWSR1* rearrangement in a neoplasm should be interpreted in the context of clinical findings, histological, immunophenotypic and molecular features. In the field of pediatric oncology, prospective studies monitoring the event‐free and overall survival in patients with various *EWSR1* rearrangements in CNS tumors are needed to better risk‐stratify and treat these patients.

Pediatric *EWSR1*‐rearranged tumors of the CNS are rare and have not been addressed comprehensively, apart from conventional Ewing sarcoma (17, 20, 21, 23, 29, 30, 35, 42, 43, 45, 56, 62, 67, 68, 81, 88, 89). The occurrence of both mesenchymal and primary glial/neuronal tumors in association with *EWSR1* rearrangements highlights their diverse biological behavior and raises interest in the identification of fusion transcripts. Here, we identified a desmoplastic small round cell tumor arising from the occipital lobe (Case 5) harboring an *EWSR1‐WT1* fusion that was confirmed by reverse transcription‐polymerase chain reaction. Because this tumor most often arises within the abdominal cavity, it was initially thought that this entity originated from primitive mesothelial progenitors; however, several cases have been reported arising from different other locations (78). The occurrence of desmoplastic small round cell tumor within the CNS is an exceptionally rare event, with only 14 reported cases (2, 11, 39, 47, 69, 77, 80, 87), 12 of which have been confirmed by molecular techniques and with only 6 verified pediatric cases (2, 11, 39) (Table 2). Our case showed atypical morphology and immunophenotype in a pattern that is similar to some of the previously reported pediatric brain cases (11, 39). Importantly, recent methylation studies have demonstrated that intracranial desmoplastic round cell tumors cluster along with their intrabdominal counterparts, supporting their shared histogenesis (39) and highlighting the importance of further molecular characterization and epigenetic profiling of primary *EWSR1‐*rearranged neoplasms of the CNS.

**Table 2:** Literature review summary of 123 *EWSR1*‐rearranged tumors with *CREB1*, *CREM*, *PLAGL1*, *PATZ1* and *WT1* fusion partners.

*EWSR1* Fusion partner	Site of origin		Age (years)	Sex	Diagnosis	Initial therapy	Outcome (mo)	Reference (#)
*CREB1* *	CNS	Dura	12	M	IMMT	Surgery (GTR)	N/A	(10)
		Brain, intraventricular	14	F		Surgery (GTR)	N/A	
		Brain, Frontal lobe	20	M		N/A	N/A	(33)
		Dura	23	F		N/A	N/A	
		Brain, intraventricular	53	F		Surgery (NTR), RT	NED (3)	(37)
		Dura	58	F	Myxoid AFH	Surgery (NTR)	AWD (3)	(26)
		Cerebellum	17	M	Extraskeletal myxoid chondrosarcoma	Surgery	AWD (36)	(82)
		Brain, parietal lobe	17	M	IMMT	Surgery (TR)	NED (2)	Case 1, present study
	Soft tissue (*n* = 31)		3‐63 (mean: 20)	1.4:1 M:F	AFH	Surgery (*n* = 22) N/A (*n* = 8) CT (*n* = 1)	NED, *n* = 15 AWD, *n* = 2 N/A, *n* = 14	(1, 6, 19, 32, 52, 61, 72, 75, 76)
	Lung		64	M	AFH	Surgery	N/A	(74)
			63	M		N/A	N/A	(72)
	GI Tract		47	F	Clear cell sarcoma‐like tumor	Surgery	N/A	(34)
			81	F	Clear cell sarcoma	CT	AWD (60)	(8)
			42	F		Surgery	N/A	
			42	F		Surgery	N/A	
			64	M		Surgery	AWD (9)	(27)
			33	M	GNET	Surgery	DOD (10)	(27)
			27	F	GNET	CT, RT	AWD (36)	(66)
	Liver		33	M	Clear cell sarcoma‐like tumor	N/A	DOD (7)	(73)
	Mediastinum		80	M	Malignant AFH	Surgery	NED (8)	(46)
	Pulmonary artery		21	F	Low‐grade myxoid sarcoma	Surgery	NED (38)	(48)
	Lymph node		8	M	AFH	N/A	N/A	(72)
	Head and neck		48	F	Clear cell odontogenic carcinoma	Surgery	N/A	(83)
*CREM*	CNS	Brain, left frontal lobe	12	F	AFH	Surgery (NTR)	AWD (28)	(38)
		Brain, right frontal lobe	18	M	IMMT	Surgery	N/A	(10)
		Dura	15	F		Surgery	NED (17)	(33)
		Brain, frontal lobe	9	M		Surgery, RT	NED (20)	(86)
		Dura	19	M	Intracranial myxoid AFH‐like tumor	Surgery (GTR), CT, RT	NED (120)	(25)
		Posterior fossa	20	F	Intracranial tumor with AFH‐like features	Surgery (GTR)	AWD (54)	Case 2, present study
	Soft tissue		20	F	Myxoid mesenchymal tumor	Surgery, RT	NED (156)	(33)
			68	F	Hyalinizing clear cell carcinoma	Surgery	NED (19)	(16)
			48	M	Ectomesenchymal chondromyxoid‐like tumor	Surgery	NED (12)	(22)
			49	F	Clear cell sarcoma	Surgery	AWD (39)	(90)
			50	M	Myxoid AFH	Surgery	NED (45)	
			54	M		Surgery	NED (51)	
			15	M	Unclassifiable spindle cell tumor	CT	DOD (18)	
			63	F		Surgery	NED (17)	
			20	F	Malignant epithelioid neoplasm	Surgery	NED (204)	(9)
			14	F	Malignant epithelioid neoplasm	Surgery	N/A	
	Pleural cavity		44	F	Malignant epithelioid neoplasm	Surgery, CT	AWD (4)	
	Kidney		29	M	Malignant epithelioid neoplasm	Surgery	N/A	
	Stomach (surrounding fundus)		25	M	Malignant epithelioid neoplasm	Surgery	N/A	
	Mesocolon		47	F	Malignant epithelioid neoplasm	Surgery	N/A	
	Adrenal gland		9	M	Malignant epithelioid neoplasm	Surgery	NED (31)	
	Lung		75	F	Hyalinizing clear cell carcinoma	Surgery	NED (8)	(16)
			47	M	Myxoid AFH	Surgery	NED (58)	(90)
	Head and neck		62	M	Hyalinizing clear cell carcinoma	Surgery	NED (5)	(16)
*PLAGL1*	CNS	Brain	2	N/A	Malignant Rhabdoid Tumor (NOS)	N/A	N/A	(54)
		Brain, frontal lobe	4	M	Glioneuronal tumor, NEC	Surgery (NTR)	NED (84)	Case 3, present study
*PATZ1*	CNS	Brain	26	F	Undifferentiated sarcoma, NOS	N/A	N/A	(12)
		Brain	21	M	Primitive neuroectodermal tumor	N/A	N/A	
		Brain	13	M	Pleomorphic xanthoastrocytoma	N/A	N/A	
		Brain	22	F	Glioma	N/A	N/A	
		Brain, lateral ventricle	32	F	Papillary glioneuronal tumor	Surgery (GTR)	NED (12)	(60)
		Cerebellum	7	F	Low‐grade glioma	Surgery (NTR)	AWD (3)	
		Brain	N/A	N/A	Pediatric high‐grade glioma, NOS	N/A	N/A	(31)
		Cervical spine	50	F	Low‐grade glial tumor	Surgery	NED (12)	(50)
		Brain	N/A	N/A	Ganglioglioma	N/A	N/A	(53)
		Brain, temporal lobe	19	F	Malignant, poorly differentiated neoplasm of the CNS	Surgery (NTR), RT	AWD (5)	Case 4, present study
	Soft tissue		16	M	Primitive neuroectodermal tumor	N/A	AWD (24)	(41)
			31	F	Malignant round and spindle cell neoplasm	Surgery, CT	DOD (5)	(18)
			53	F		Surgery, CT	NED (3)	
			1	M	Malignant spindle cell neoplasm	N/A	N/A	(84)
			32	M	Ewing or Ewing‐like sarcoma	N/A	N/A	
			46	F	Unclassified malignant neuroectodermal tumor	N/A	N/A	
			69	M	Malignant spindle cell tumor w/hemangioma‐like features	N/A	N/A	
			11	F	Undifferentiated round cell sarcoma	N/A	N/A	(12)
			35	F	Undifferentiated round cell sarcoma	CT	DOD (30)	
			53	M	Soft tissue myoepithelial neoplasm	N/A	DOD (2)	
			60	M	Undifferentiated sarcoma, NOS	N/A	N/A	
			81	F	Undifferentiated low‐grade sarcoma	N/A	NED (19)	
			52	F	Polyphenotypic round cell sarcoma	Surgery, CT, RT	NED	(49)
			37	M	Round cell sarcoma	N/A	N/A	(79)
			6	M	Round cell sarcoma	N/A	N/A	
			54	M	Round cell sarcoma	N/A	N/A	
	Lung		59	M	Undifferentiated sarcoma, NOS	N/A	N/A	(12)
	Mediastinum		57	M	Malignant epithelioid spindle cell neoplasm	N/A	N/A	(84)
	Head and neck		19	F	Alveolar rhabdomyosarcoma	N/A	N/A	(12)
	N/A		26	N/A	Glioneuronal tumor	N/A	N/A	(4)
*WT1* ^†^	CNS	Posterior fossa	24	M	Desmoplastic small round cell tumor	Surgery (STR), CT, RT	NED (36)	(77)
		Brain, temporal lobe	6	F		Surgery (GTR), CT, RT	NED (18)	(11)
		CPA	37	M		Surgery (GTR), CT, RT	DOD (24)	(47)
		Posterior fossa	39	M		CT, RT	AWD (27)	
		Brain, suprasellar	27	M		Surgery (NTR), RT	DOD (20)	(69)
		Intratemporal fossa	6	M		N/A	N/A	(2)
		CPA	37	M		CT, RT	DOD (32)	
		Brain, temporal lobe	13	M		Surgery (GTR), RT	NED (16)	(39)
		Brain, occipital lobe	6	M		N/A	N/A	
		Cerebellum	25	M		Surgery (GTR)	DOD (1)	
		Brain, parietal lobe	11	M		Surgery (GTR), CT, RT	NED (13)	
		Brain, frontal lobe	8	M		Surgery (GTR), CT, RT	NED (96)	
		Brain, occipital lobe	6	M		Surgery (STR)	N/A	Case 5, present study
		Dura of cauda equina	34	M	Low‐grade small round cell tumor	Surgery (STR)	AWD (9)	(80)

Abbreviations: AFH = angiomatoid fibrous histiocytoma; AWD = alive with disease; CNS = central nervous system; CPA = cerebellopontine angle; CT = chemotherapy; DOD = died of disease; GNET = gastrointestinal neuroectodermal tumor; GTR = gross total resection; IMMT = intracranial myxoid mesenchymal tumor; N/A = not available; NEC = not elsewhere classified; NED = no evidence of disease; NOS = not otherwise specified; NTR = near‐total resection; RT = radiation therapy; STR = subtotal resection.

* Tumors harboring a *EWSR1‐CREB1* fusion gene with a diagnosis of primary pulmonary myxoid sarcoma were excluded from this review as these represent well‐defined entities.

^†^ Only primary CNS desmoplastic small round cell tumors were included in this review.

We identified two primary brain neoplasms with *EWSR1* fusions involving partners from the CREB family of transcription factors, expanding the existing literature on a broadly diverse group of neoplasms (3, 6, 7, 8). The *EWSR1‐CREB1* fused tumor (Case 1), showed a morphologic spectrum nearly identical to several other intracranial myxoid mesenchymal tumors, including a prominent vascular component, scattered blood‐filled cystic spaces and focal amianthoid collagen fiber deposition (10, 25, 33, 38). A review of the literature shows this pattern overlaps myxoid angiomatoid fibrous histiocytoma, as it has been described in a number of *CREB1* and *CREM* fused tumors of the CNS arising from extra‐parenchymal/dural locations as well as parenchymal sites (Table 2).

Case 2 in our series, despite the presence of an *EWSR1‐CREM* fusion was difficult to categorize because of its lack of resemblance to either intracranial myxoid mesenchymal tumors or angiomatoid fibrous histiocytoma, similar to a recently reported case of pediatric intracranial *EWSR1‐CREM* fused tumor (86). Classification of our tumor was complicated by the striking epithelioid morphology, high proliferative index, absence of myxoid or angiomatoid features and further progression to pelvic bone metastasis. However, case 2 diffusely expressed GLUT‐1 in a peculiar paranuclear (Golgi‐type) pattern, a marker that has been reported positive in other intracranial myxoid tumors harboring the same genetic alteration (10, 86). Although uncommon in this location, a clear cell sarcoma of soft parts was initially entertained but ultimately excluded, given the negative melanocytic markers. The question remains if this case may be related to the group of high‐grade neuroectodermal tumors of the gastrointestinal tract with absent melanocytic differentiation which have an uncertain relationship with clear cell sarcoma of soft parts (14, 63); however, *EWSR1‐CREM* rearrangements have never been reported in these rare tumors (90), except by a presumptive case in which the fusion gene was not confirmed (57). Interestingly, a recent series reported two unclassifiable soft tissue tumors harboring a *EWSR1‐CREM* fusion with minimal morphologic and immunophenotypic overlap with our cases, one of them arising in a young patient who died following an aggressive course and multiple liver metastases (90). Furthermore, a recent study described a group of distinctive malignant epithelioid tumors with *EWSR1/FUS‐CREB* fusions and predilection for mesothelial‐lined cavities; including seven *EWSR1‐CREM* fused tumors (two of them in children); however, no CNS involvement was identified in this series (9). Whether these *EWSR1‐CREM* neoplasms arising at various sites are histogenetically related would be worth investigation with methylation studies and highlights their increasingly diverse morphologic spectrum (Table 2).

Evidently, our understanding of the so‐called intracranial myxoid mesenchymal tumors continues to evolve as more cases sharing similar features are being documented. The assertion that *EWSR1‐CREM* rearrangements have never been reported in “classic” angiomatoid fibrous histiocytomas was, until very recently, considered accurate. Nonetheless, this notion was recently contested by Konstantinidis *et al*, who reported two pediatric cases of “classic” intracranial angiomatoid fibrous histiocytomas lacking myxoid features and harboring *EWSR1‐ATF1* and *EWSR1‐CREM* fusions, respectively (38). Furthermore, it is well‐known that angiomatoid fibrous histiocytoma features a wide‐ranging morphological and biological spectrum (24, 25, 59, 70, 86). In a recent study, both *EWSR1‐CREB1* (n = 8) and *EWSR1‐CREM* (n = 3) fusions were found in extracranial angiomatoid fibrous histiocytomas featuring a wide and variable spectrum of ages, locations and histology; suggesting a closer relationship between angiomatoid fibrous histiocytomas and the so‐called CREB‐related intracranial myxoid neoplasms (90).

In addition to a morphology, the limited outcome data available in the literature suggests that CREB family fused tumors behave similarly if they arise in the CNS or soft tissue locations (Table 2). Of the CNS cases with outcome data, all eight were alive at the time of follow‐up with half having no evidence of disease (Table 2). Among patients with soft tissue *CREB1* and *CREM* family fused tumors with follow‐up data (n = 29), most (n = 24) had no evidence of disease following therapy, four were alive with disease and only one was dead of disease (Table 2). Taken altogether, our findings along with the review of the literature are in favor of these lesions being considered as a continuum rather than separate or novel entities, as it was originally suggested (33). Larger studies comparing CNS and soft tissue counterparts using methylation analysis with longer follow‐up data as well as fusion gene functional analysis would be helpful.

Case 3 illustrates an extremely unusual *EWSR1‐PLAGL1*rearranged glioneuronal tumor with variable immunophenotype. *EWSR1‐PLAGL1* is an exceptionally rare fusion gene that, to the best of our knowledge, has been succinctly reported only once, in the setting of an INI‐1 deficient pediatric atypical teratoid rhabdoid tumor (54) (Table 2). Unfortunately, this reported case was described as part of a large‐scale molecular study and no additional morphologic or immunophenotypic details were provided. Our case was morphologically heterogeneous without rhabdoid features and no large deletions involving *SMARCB1* and *SMARCA4* were detected by single nucleotide polymorphism chromosomal microarray. This was further supported by preserved immunohistochemistry for INI‐1 and BRG‐1, virtually excluding the possibility of an atypical teratoid rhabdoid tumor. Furthermore, atypical teratoid rhabdoid tumors tend to show aggressive behavior and overall unfavorable prognosis; in contrast, this tumor presented with an indolent course and our patient is enjoying a favorable outcome after 84 months of follow‐up following near total resection. Given that this tumor’s methylation profile did not classify with known CNS tumors, the best diagnosis based on clinical, morphologic immunohistochemical features is most consistent with a glioneuronal tumor not elsewhere classified. Collecting whole‐ genome DNA methylation data from these rare entities would be of help in defining new methylation classes for further diagnostic refinement (36). Recently, two pediatric CNS tumors have been identified with high‐level amplification of chromosome 6q24.2 corresponding to a common region, including *PLAGL1*, potentially representing new entities (13). The *PLAGL* genes (particularly *PLAGL2*), have been previously implicated as drivers in gliomas (54), promoting progenitor cell self‐renewal and proliferation as well as modulation of cellular differentiation. We could speculate that the observed biphasic pattern may be related to a “dual effect” of *PLAGL1* in promoting the proliferation of primitive cells while modulating glioneuronal differentiation, although this hypothesis requires further study.

One of the most diagnostically challenging tumors in our cohort is Case 4, a poorly differentiated neoplasm showing a negative result by FISH for *EWSR1* rearrangement. However, targeted RNA‐sequencing revealed an underlying *EWSR1‐PATZ1* fusion gene. This rare rearrangement results from a submicroscopic paracentric inversion, which accounts for the negative results, as FISH studies may fail to detect cryptic rearrangements or alterations resulting from small intrachromosomal inversions, leading to equivocal gaps between the split probes (12, 60). Interestingly, *EWSR1‐PATZ1* fusions have been reported in small round cell sarcomas (12, 18, 84) and more recently, in association with low‐ and high‐grade glioneuronal neoplasms (28, 31, 53, 60). These associations are particularly relevant here, as the same fusion gene has been described in tumors of apparently different lineages (mesenchymal vs. neuroectodermal) that are not possible to assess in our case, given its poorly differentiated nature. As such, we failed to confirm its histogenesis following a broadly negative panel of immunostains, including neuronal, glial, epithelial and mesenchymal markers (Table S2). Clearly, our understanding of poorly differentiated tumors is still limited even after characterization of fusion partners and methylation status. Additional studies examining tumors with rare fusions by methylation status may help define new diagnostic entities and are useful for comparing tumors with the same fusion from different locations (soft tissue vs. CNS).

Similar to Cases 1 and 2, Case 4 harboring *EWSR1‐PATZ1* rearrangement represents another instance of identical fusion genes shared between soft tissue tumors and CNS tumors (See also Table 2). Other examples are represented by the group of *BCOR‐*internal tandem duplication neuroepithelial tumors (91) and by previously discussed *EWSR1‐CREB* tumors. Often, these entities may show morphologic and immunohistochemical differences disclosing a different histogenesis. Some have proposed that identical gene fusions can be associated with different histotypes caused by the local effects of different anatomic locations in otherwise similar progenitor cells. Conversely, others suggest that the same genetic alteration among different progenitor cells (possibly mesenchymal stem cells) could lead to differential transcriptional activation (64, 71). We hypothesize that the same fusion gene may result in apparently different phenotypes depending on the degree of cellular differentiation in which the genetic alteration occurs, as already reported in experimental models of rhabdomyosarcomas and undifferentiated sarcomas by Rubin *et al* (55). Methylation studies in these tumors may help resolve some of these issues and should be performed on larger cohorts.

In conclusion, we describe five rare primary CNS pediatric tumors exhibiting a broad range of morphologic and biologic features in the setting of various confirmed *EWSR1*‐non‐ETS gene fusions. Our series highlights the importance of routine characterization of fusion partners beyond conventional *EWSR1*‐FISH studies, combined with histopathology and immunohistochemistry, to define these entities as they can show overlapping molecular signatures, a broadly variable biologic behavior and divergent therapeutic approaches. Larger prospective studies that incorporate morphologic and molecular features, methylation signatures, therapeutics and outcome data of pediatric CNS tumors with *EWSR1* rearrangements are needed to further delineate the biology of these rare tumors in comparison to their extracranial soft tissue counterparts. These studies are also crucial to clarify ongoing clinical questions and to determine the prognostic implications of histotype, anatomical location and fusion partners.

## Conflict of Interest

The authors declare that they have no conflict of interest to disclose for the current study.

## Author Contributions

LFS and RA designed the study. OLN and BC collected data and wrote the original manuscript and further revisions. LFS, AZ, MML, YZ and EM performed molecular assays and analyzed the molecular data. MS, MPN, SMK, TMP, SaRo, RA and SaRa were responsible for histopathological evaluation. KMB and AB were responsible for clinical assessment. All authors made intellectual contributions, provided critical revision of the manuscript and approved the final manuscript.

## Supporting information


**Figure S1.** *EWSR1‐CREM* fused tumor (case 2).

**Figure S2.** *EWSR1‐PLAGL1* fused tumor (case 3).

**Figure S3.** *EWSR1‐PATZ1* fused tumor (case 4).

**Table S1.** Summary of antibodies used for immunohistochemistry.

**Table S2.** Immunohistochemical profile of 5 *EWSR1*‐rearranged primary CNS neoplasms.

**Table S3.** Detailed molecular features.

## Data Availability

Data sharing is not applicable to this article as no datasets were generated or analyzed during the current study
