# Supplementary figures and images for: The spectrum of rare central nervous system (CNS) tumors with EWSR1‐non‐ETS fusions: experience from three pediatric institutions with review of the literature
Source: Brain Pathol. 2020 Nov 6;31(1):70–83. doi: 10.1111/bpa.12900 (PMC8018079; doi:10.1111/bpa.12900)

# EWSR1-CREM fused tumor (case 2)

**A**

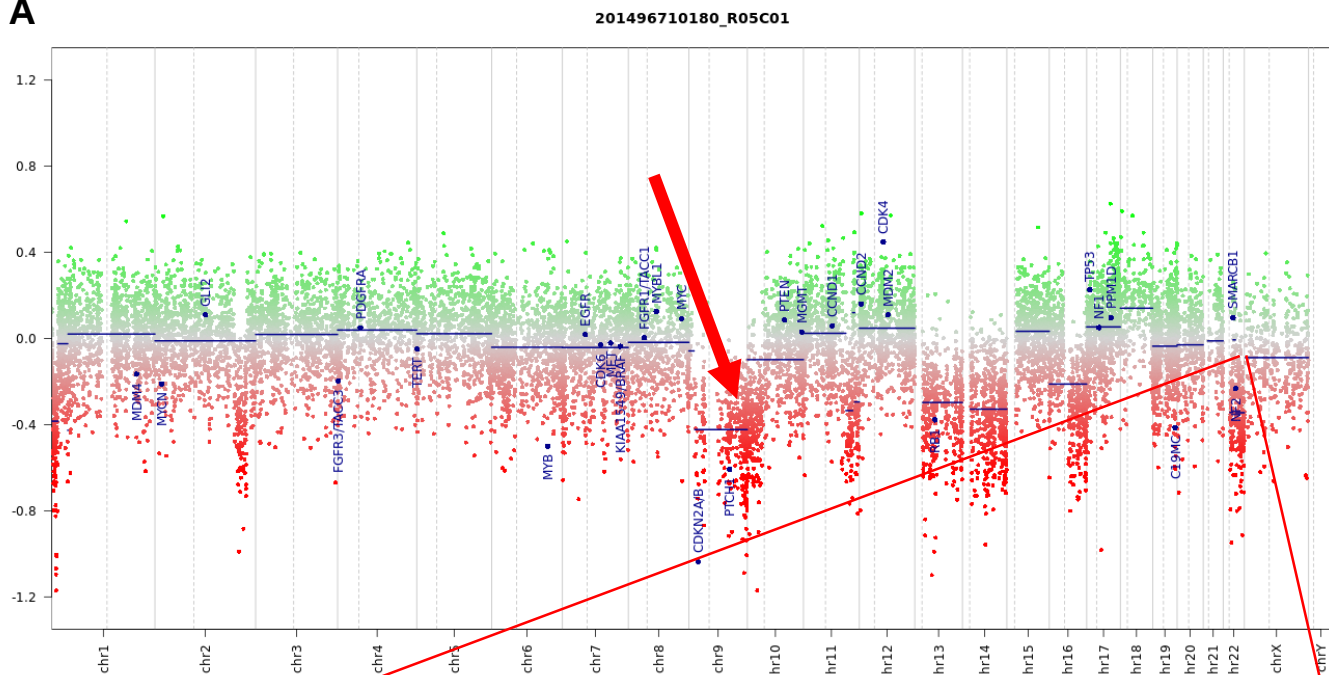

**B**

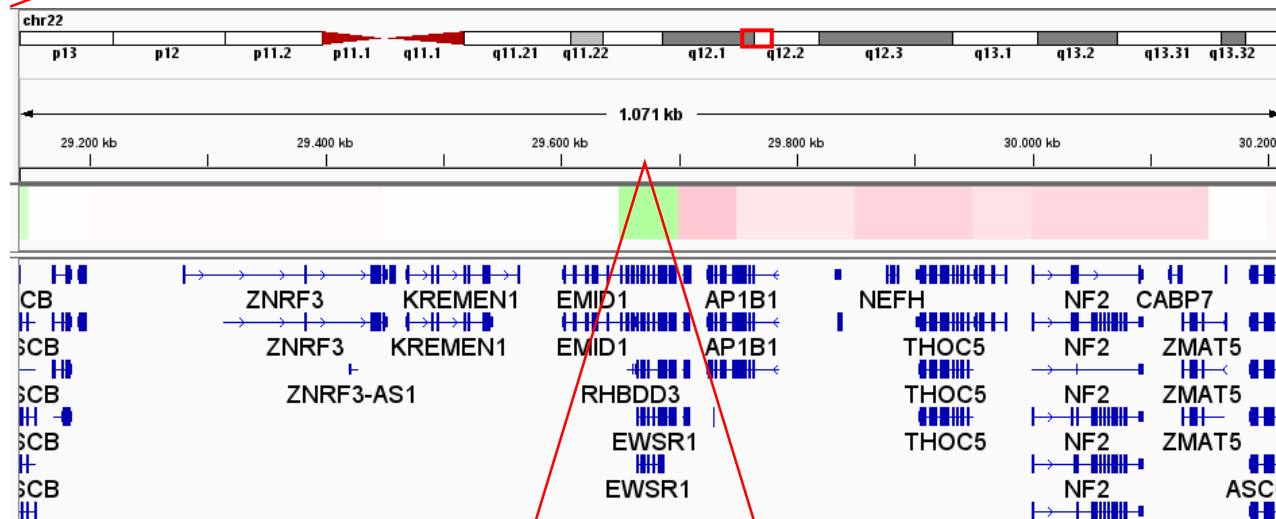

Supplement: Supplementary file 1 — Figure S1. EWSR1‐CREM fused tumor (case 2). [file BPA-31-70-s005.pdf]

***EWSR1-PLAGL1* fused tumor (case 3)**

**A**

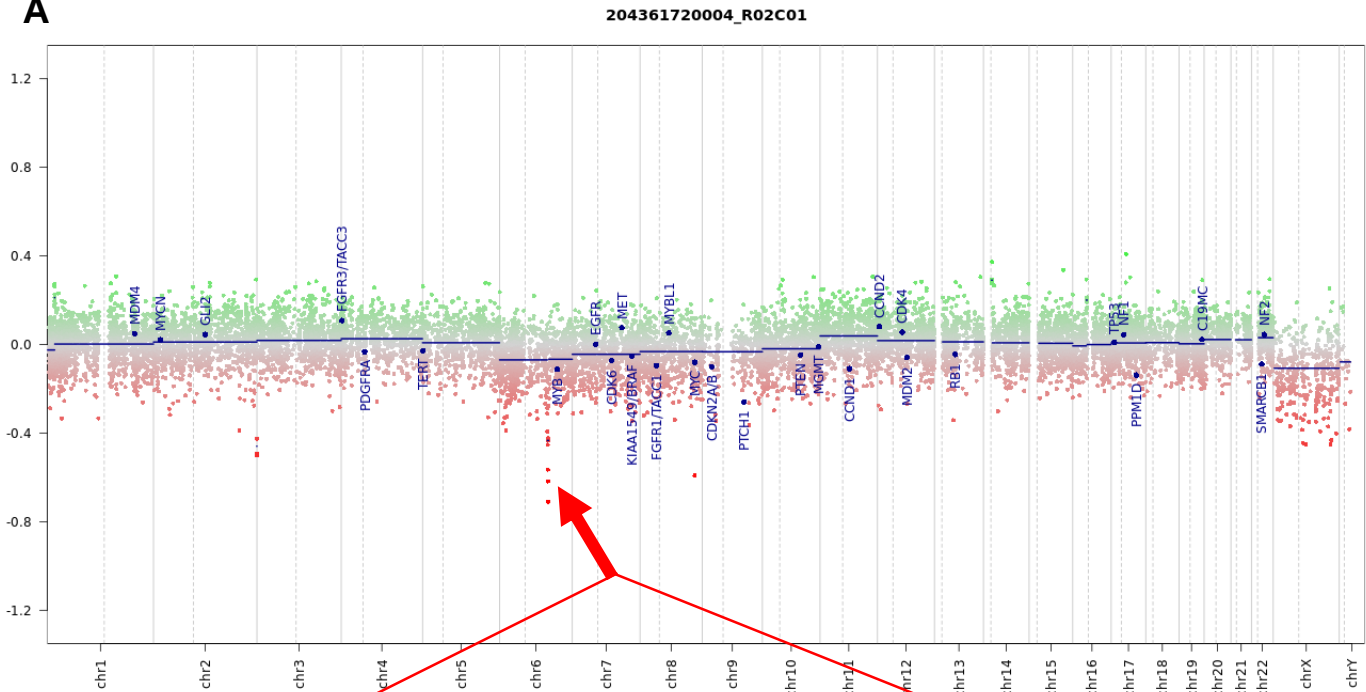

**B**

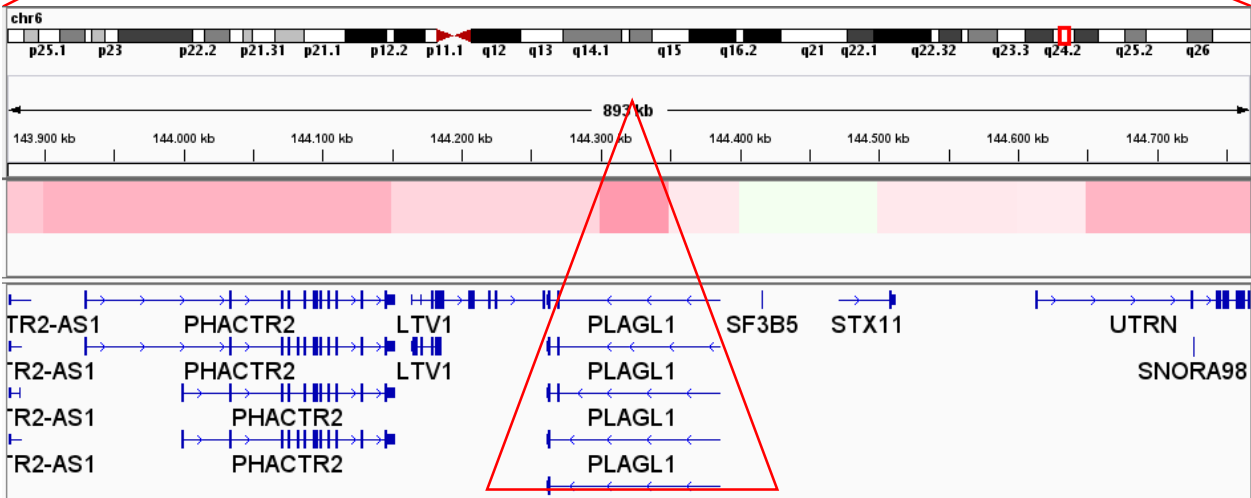

Supplement: Supplementary file 2 — Figure S2. EWSR1‐PLAGL1 fused tumor (case 3). [file BPA-31-70-s004.pdf]

EWSR1-PATZ1 fused tumor (case 4)

A

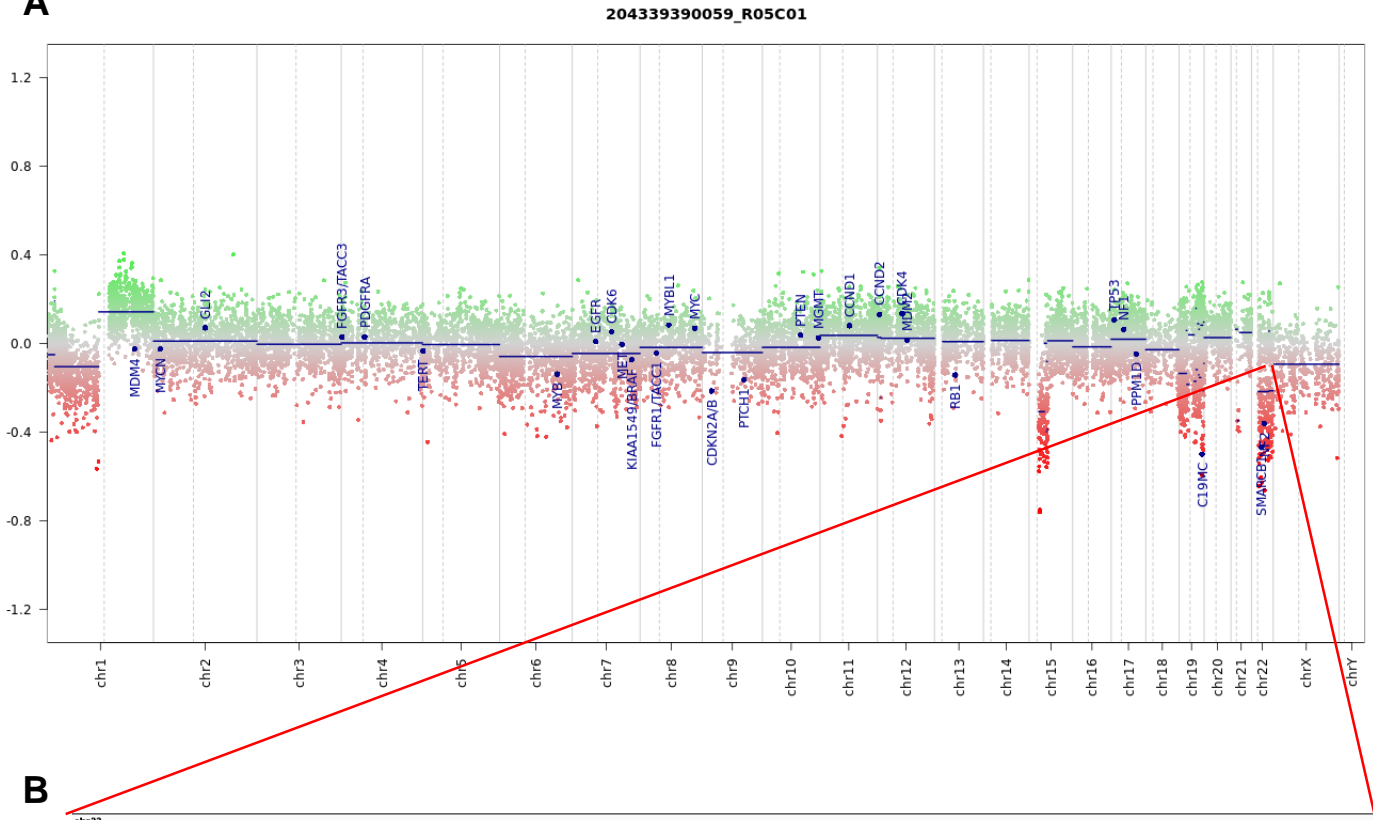

B

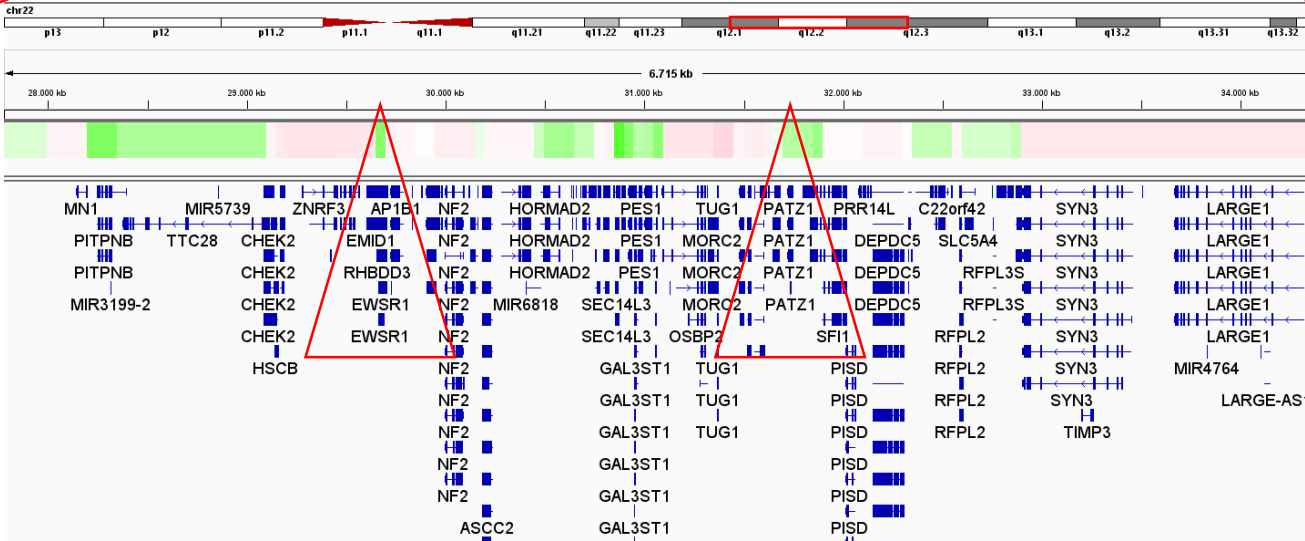

Supplement: Supplementary file 3 — Figure S3. EWSR1‐PATZ1 fused tumor (case 4). [file BPA-31-70-s003.pdf]
