# Supplementary material for: The spectrum of rare central nervous system (CNS) tumors with EWSR1‐non‐ETS fusions: experience from three pediatric institutions with review of the literature
Source: Brain Pathol. 2020 Nov 6;31(1):70–83. doi: 10.1111/bpa.12900 (PMC8018079; doi:10.1111/bpa.12900)
Supplement: Supplementary file 4 — Table S1. Summary of antibodies used for immunohistochemistry. [file BPA-31-70-s002.docx]

| **Supplemental Table 1. Summary of antibodies used for immunohistochemistry** | | |
| --- | --- | --- |
| **Antibody** | **Clone** | **Dilution** |
| CD99 | 12E7, Dako, Glostrup, Denmark | 1:100 |
| S-100 | Polyclonal, Leica; Newcastle, United Kingdom | RTU |
| Cytokeratin AE1/AE3 | AE1/AE3, Dako; Glostrup, Denmark | 1:100 |
| Synaptophysin | 27G12, Leica; Newcastle, United Kingdom | 1:100 |
| ATRX | Polyclonal, Sigma-Aldrich; Darmstadt, Germany | 1:100 |
| GFAP | 6F2, Dako; Glostrup,Denmark | 1:400 |
| p53 | DO-7, Dako; Glostrup,Denmark | 1:100 |
| IDH-1 | H09, Dianova; Hamburg, German | RTU |
| Anti-Human Epithelial Membrane Antigen | E29, Dako; Glostrup, Denmark | 1:200 |
| Anti-Human Muscle Actin | HHF35, Dako; Glostrup, Denmark | 1:800 |
| SOX9 | AB5535, Chemicon | 1:1000 |
| RTU = ready to use | | |
