# Supplementary material for: The spectrum of rare central nervous system (CNS) tumors with EWSR1‐non‐ETS fusions: experience from three pediatric institutions with review of the literature
Source: Brain Pathol. 2020 Nov 6;31(1):70–83. doi: 10.1111/bpa.12900 (PMC8018079; doi:10.1111/bpa.12900)
Supplement: Supplementary file 5 — Table S2. Immunohistochemical profile of 5 EWSR1‐rearranged primary CNS neoplasms. [file BPA-31-70-s006.docx]

| **Supplemental Table 2. Immunohistochemical profile of 5 *EWSR1*-rearranged primary CNS neoplasms** | | | | | |
| --- | --- | --- | --- | --- | --- |
| **Case** | **Diffusely positive** | **Focally positive** | **Negative** | **Ki67(%)** | **Other** |
| 1 | N/A | CD99, S100, GLUT1, EMA | GFAP, Synaptophysin, OLIG2, SOX10, Desmin, SSTR2, BRAF V600E, CA-IX, Inhibin, ALK-1, CD31, CD34, ERG, SMA, STAT6, SOX9 | 4 | N/A |
| 2 | GLUT1 (paranuclear/Golgi-like), CD99 | EMA, GFAP, Synaptophysin | OLIG2, Desmin, S100, HMB45, Melan-A | 50 | N/A |
| 3 | Synaptophysin, Neurofilament protein, GFAP, NeuN | EMA, S100 | CD99, Cytokeratin AE1/AE3, SMA | 5-10 | INI-1 retained; BRG-1 retained |
| 4 | N/A | N/A | GFAP, OLIG2, S100, SOX10, Synaptophysin, Desmin, SMA, CD68, EMA, Cytokeratin AE1/AE3, PAX8, ER, TLE, ALK-1, BCOR, STAT6, IDH-R132H, Neurofilament | 70 | INI-1 retained, BRG-1 retained, ATRX retained, p53 (lower to moderate expression within tumor) |
| 5 | Desmin | N/A | MyoD1, Cytokeratin AE1/AE3 | N/A | INI-1 retained; BRG-1 retained |
| Abbreviations: N/A= not applicable, GFAP= Glial fibrillary acidic protein, NeuN= Neuronal nuclei, CNS= Central nervous system. | | | | | |
