# Supplementary material for: The spectrum of rare central nervous system (CNS) tumors with EWSR1‐non‐ETS fusions: experience from three pediatric institutions with review of the literature
Source: Brain Pathol. 2020 Nov 6;31(1):70–83. doi: 10.1111/bpa.12900 (PMC8018079; doi:10.1111/bpa.12900)
Supplement: Supplementary file 6 — Table S3. Detailed molecular features. [file BPA-31-70-s001.docx]

| **Supplemental Table 3. Detailed molecular features** | | | | | | | | |
| --- | --- | --- | --- | --- | --- | --- | --- | --- |
| **Case** | ***EWSR1*-FISH** | ***EWSR1* Breakpoint (NM_005243.3)** | | **3' Gene Partner** | | | | **Detection Method** |
|  |  | **Included Exons** | **Chromosomal breakpoint (GRCh37)** | **Gene** | **Transcript** | **Included Exons** | **Chromosomal breakpoint (GRCh37)** |  |
| 1 | Positive | 1-7 | chr22:29,683,123 | *CREB1* | NM_134442.4 | 7-9 | chr2:208,439,996 | NGS |
| 2 | Positive | 1-13 | chr22:29,693,940 | *CREM* | NM_181571.2 | 6-8 | chr10:35,477,128 | NGS |
| 3 | Positive | 1-8 | chr22:29,684,775 | *PLAGL1* | NM_006718.4 | 7 | chr6:144,263,800 | NGS |
| 4 | Negative* | 1-8 | chr22:29,684,775 | *PATZ1* | NM_014323.2 | 1-5** | chr22:31,740,629 | NGS |
| 5 | Positive | 1-7 | chr22:29,683,123 | *WT1* | NM_024426.5 | 8-10 | chr11:32,414,301 | RT-PCR |
| Abbreviations: NGS = Next generation sequencing-based technique, RT-PCR= Reverse transcription polymerase chain reaction, N/A = not available. | | | | | | | | |
| **EWSR1* rearrangement confirmed by NGS.  **Fusion occurs in the middle of exon 1 and also contains a 21 base pair linker that maintains reading frame: 5' CCATAGAAGAGACCATGGGAG. | | | | | | | | |
